# Effect of enhancing village health volunteer ability to promote engaged community-based interprofessional education

**DOI:** 10.5116/ijme.67ab.5af9

**Published:** 2025-03-26

**Authors:** Kitsarawut Khuancharee, Chawin Suwanchatchai, Suthee Rattanamongkolgul

**Affiliations:** 1 Department of Preventive and Social Medicine, Faculty of Medicine, Srinakharinwirot University, Nakhon Nayok, Thailand

**Keywords:** Village health volunteer, VHVs’ abilities program, associate teachers, community-based interprofessional education

## Abstract

**Objectives:**

To evaluate the impact of enhancing village health volunteers' (VHVs) abilities to promote engaged community-based interprofessional education (CBIPE).

**Methods:**

A single-group pre-posttest design was implemented with 100 VHVs enrolled in a VHVs' abilities program. The program consisted of a two-day workshop that included five key sessions: a 30–40-minute lecture, demonstration and replay, 15–30-minute information sharing and communication, 60–150-minute discussion and practice, and 30-minute feedback. Of the participants, 83 VHVs completed the course and provided data for analysis. Outcomes measured included attitude and motivation towards associate teachers, self-esteem, community diagnosis knowledge, and course satisfaction. Repeated-measures ANOVA was used to analyze changes in competency scales over time.

**Results:**

A significant increase in community diagnosis knowledge was observed post-intervention (mean difference = 26, 95% CI = 24–28; p < 0.001). Significant improvements were also seen in attitude (mean difference = 1.00, 95% CI = 0.96–1.04; p < 0.001), motivation (mean difference = 0.92, 95% CI = 0.86–0.97; p < 0.001), communication (mean difference = 0.95, 95% CI = 0.92–0.97; p < 0.001), and systems thinking (mean difference = 0.98, 95% CI = 0.97–1.00; p < 0.001). No significant change was observed in the active listening scale (p = 0.104). VHVs expressed high satisfaction with the program, with an average score of 4.13 ± 0.76.

**Conclusions:**

VHVs' abilities programs effectively enhance knowledge and improve VHVs' competencies. Ongoing training for associate teachers is essential to support engaged learning and CBIPE field practice for medical students.

## Introduction

Community-based education (CBE) is a highly effective approach for developing collaborative skills among health profession students worldwide.^1^^,^^2^ Community-based interprofessional education (CBIPE) is a collaborative learning approach that occurs in a community setting. It involves students from different backgrounds working together to learn and carry out a community diagnosis. This method promotes teamwork and offers opportunities for the practical application of knowledge in real-life situations.^3^^,^^4^ The objective is to enable learners to gain hands-on experience in direct professional collaboration and enhance their understanding of their respective fields within a community context. Previous studies have demonstrated that CBIPE is an effective approach to improving the cooperative abilities of health professional students.^5^^-^^10^ CBIPE programs provide opportunities for active engagement of both students and community members in various learning activities throughout the educational experience and practical fieldwork,^11^^,^^12^ including village health volunteers (VHVs) and local wisdom. By involving these stakeholders in the educational process, CBIPE programs enrich the learning experience, promote community empowerment, and facilitate the exchange of valuable knowledge and perspectives between students and community members. This collaborative approach ensures that learning is not confined to the classroom but extends into real-world contexts, where students can apply their skills and knowledge to address genuine community needs. VHVs have been instrumental in Thailand's primary healthcare system, serving as vital contributors to healthcare delivery. VHVs have helped mitigate the healthcare resource crisis by delivering essential care at the household and community levels, reducing healthcare inequality for the vulnerable population.^13^^,^^14^ VHVs conduct health surveys and administer health promotion and disease prevention campaigns through home visits and health education sessions at the district and subdistrict levels.^15^^,^^16^

During one week of field practice in the CBIPE program, medical students undergo practical training in rural communities. They are divided into 12 groups, with each group being assigned to a different village within the rural area. Importantly, each group is supported by VHVs who play a crucial role in facilitating the field practice activities. VHVs provide support and guidance to medical students throughout their fieldwork experience. VHVs help students navigate the community dynamics, provide insights into local health issues, and facilitate interactions with community members. Additionally, VHVs may assist students in conducting health assessments, organizing health promotion activities, and addressing community health needs identified during the field practice. By working alongside VHVs, medical students gain valuable exposure to community-based healthcare delivery and interprofessional collaboration. This hands-on experience allows them to apply theoretical knowledge in real-world settings, develop essential clinical skills, and cultivate a deeper understanding of the social determinants of health within rural communities. However, the field experience report revealed that more than half of VHVs encounter challenges that hinder their ability to perform effectively, such as not being fully integrated into the professors and medical students, inadequate resources, and knowledge to identify health problems and community diagnosis, and low confidence. Even though VHVs were trained to provide peer support to medical students during community health surveys, VHVs still lacked confidence in their community diagnosis knowledge and skills. This lack of confidence can lead to ineffective support for medical students participating in CBIPE field practice. To enhance VHVs' abilities in promoting engaged CBIPE field practice among medical students, the study aimed to evaluate the effectiveness of a VHVs' abilities program. This program likely focused on empowering VHVs to improve their self-confidence in their knowledge and skills related to community diagnosis and other relevant areas.

## Methods

### Study design

A single-group, non-randomization with a pre-post-posttest design was conducted to evaluate the effectiveness of the VHVs’ abilities program in promoting engaged CBIPE field practice for medical students. The study was conducted in a rural area located in Ban Phrao subdistrict, Ban Na district, Nakhon Nayok province, Thailand. The convenience sampling technique was used. The study population consisted of 100 VHVs who met the following inclusion criteria: having more than one year of experience and willingness to participate in the program. VHVs being involved in an accident, sustaining injuries requiring treatment, or surgery, or being hospitalized for more than one month were excluded from the study. VHVs who resigned from their position as public health volunteers during the research data collection period were also excluded from the study. This study was approved by the Ethics Committee of Srinakhirinwirot University and the Ethics Committee of Nakhon Nayok Provincial Public Health Office (Approval number NPHO-2023-027). Informed written consent was obtained from all the participants before their involvement in the study.

### Program intervention

The principal investigator (KK) applied Bloom's learning theory 17 as the program framework to enhance the VHVs' abilities program for promoting engaged CBIPE field practice for medical students. Bloom's learning theory categorizes individual learning objectives into three domains: cognitive domain – teaching to achieve community diagnosis knowledge; affective domain – the attitude of learners toward associate teachers; and psychomotor domain – the ability to practice after learning. The VHVs' abilities program consisted of five modules, each lasting 90-120 minutes, led by the principal investigator (KK) and research members (CS and SR). These modules were conducted during a two-day intensive training workshop. After the completion of the workshop, the effectiveness of the VHVs' abilities program was evaluated through a follow-up period of 12 weeks.

Session 1 (Day 1/1) commenced with a 30-minute segment dedicated to addressing gaps in the VHVs' knowledge regarding the intervention and motivations to enhance their competency. This segment aimed to encourage VHVs to share their experiences, fostering a relationship built on trust. Following this, the subsequent 120 minutes of the session provided general information related to CBIPE. Additionally, the session covered the roles and responsibilities of associate teachers in facilitating CBIPE activities. The session also provided opportunities for interaction and communication with peers.

Session 2 (day 1/2) began with 30 minutes of peer-mediated activities to encourage the VHVs to share their previous experiences. The small group discussion and training then focused on peer support and empowerment for 40 min. The VHVs also learned and practiced the concept of active listening, communication skills, self-esteem, and how to work with peers in different situations for 120 min. Finally, the session concluded with 30 minutes dedicated to reflection and small group discussions.

Session 3 (Day 2/1) focused on information-sharing and communication, starting with a 15-minute segment of peer activities designed to encourage VHVs to share their previous experiences and challenges related to obtaining and disseminating information. 15-minute small group discussion aimed at reviewing previous knowledge regarding the concepts of community diagnosis. Over a span of 150 minutes, VHVs were divided into 12 groups and assigned to work collaboratively to explore the context of rural communities using seven community diagnosis tools, including geo-social mapping, community organization, community health system, community calendar, community history, genogram-genealogical diagram, and life history.

Session 4 (Day 2/2) was conducted in small groups, with VHVs assigned to train with their peers on identifying health problems using the community syndrome approach. This approach involved analyzing health issues from various perspectives, including biological factors, social determinants, and community regulations, over a duration of 60 minutes. Each VHV was required to interact with another to develop their abilities and relationships. The small group discussion and training then focused on feedback delivery from peers for about 30 min.

Session 5 (day 2/3) was carried out in small groups, with VHVs assigned to train with peers on identifying risk factors using a web of causes as a framework to explain the interrelationship between several disease-causing or risk factors that contribute to the cause of a specific medical condition in rural communities for 60 min. The small group discussion and training then focused on feedback delivery from peers for about 30 min. Subsequently, each VHV was evaluated for abilities, and follow-up was conducted at 1, and 12 weeks after training. At the end of the study, research questionnaires and focus group discussions (FGDs) were conducted to evaluate the program’s feasibility and gather feedback from participants regarding their experiences and perceptions of the program's effectiveness.

### Data collection

In the implementation and evaluation of the program, data collection was conducted by the research team from the baseline (Before starting the program, 1 week on 27 October 2023) to 1 (11 November 2023), and 12 weeks (3 February 2024) after the program at Ban Phrao community hall, Ban Na district, Nakhon Nayok province, Thailand. Before implementing the program, all participants were assessed for VHV abilities. At the end of the program, all participants were given the post-test survey following the conclusion of the program. One supervisor (KK) was recruited and assessed the community diagnosis and system thinking skills by analytic rubric performance assessments throughout the direct observation of VHVs activities on day 2 (after initiating the program) and 12 weeks after completing the program.

The questionnaire used in the study comprised six parts: demographics, attitudes, motivation, knowledge, self-esteem and skills, and satisfaction with the program. The content validity of the questionnaire was evaluated using the Content Validity Index (CVI) by three experts. The CVIs of knowledge, attitudes, motivation, self-esteem, skills, and satisfaction with program items were 0.90, 0.89, 0.85, 0.80, and 0.79, respectively. The first part was 10 questions related to basic demographic information, including age, gender, educational background, main occupation, marital status, monthly income, health status, body mass index, years of experience as a VHV, and personal skills and abilities.

Attitude towards associate teachers was measured using ten items. The attitudes section assessed participants' attitudes towards various aspects related to their role as VHV, including interprofessional education, community engagement, and collaboration with medical students. The item was a four-point Likert scale with responses ranging from 1 (completely disagree), 2 (disagree), 3 (agree), to 4 (completely agree). The attitude scale demonstrated excellent internal consistency reliability, with a Cronbach's alpha value of 0.96.

The motivation assessment in the role of VHV was conducted using a self-reported questionnaire consisting of five items. The item was a five-point Likert scale with responses ranging from 1 (completely disagree), 2 (disagree), 3 (unsure), 4 (agree), to 5 (completely agree). The possible scores on the motivation scale ranged from 3.41 to 5.00 points, with higher scores indicating higher levels of motivation in the role of VHV. The motivation scale demonstrated good internal consistency reliability, with a Cronbach's alpha value of 0.94.

The self-esteem and skills assessment, which likely encompassed dimensions such as active listening, communication, and analytic and system thinking, was measured using a self-reported questionnaire consisting of five items. The item was a five-point Likert scale with responses ranging from 1 (completely disagree), 2 (disagree), 3 (unsure), 4 (agree), to 5 (completely agree). The possible scores on the self-esteem and skills scale ranged from 3.41 to 5.00 points, with higher scores indicating higher perceived performance in the assessed skills. The self-esteem and skills scale demonstrated excellent internal consistency reliability, with a Cronbach's alpha value of 0.97.

Community diagnosis knowledge was measured through analytic rubric performance assessments, conducted via direct observation of VHVs’ activities on day 2 (after initiating the program) and 12 weeks after completing the program. The community diagnosis knowledge score was calculated as the sum score of responses, ranging from 0 to 100. Scores ranging from 80-100 indicated a high level of knowledge, while scores ranging from 60 to 79 and 0 to 59 indicated a moderate to low level of knowledge, respectively. The final part of the questionnaire measured satisfaction with the program, utilizing ten items rated on a five-point Likert scale. Participants were asked to rate their satisfaction levels, ranging from 1 (very dissatisfied), 2 (dissatisfied), 3 (neutral), 4 (satisfied), to 5 (very satisfied), regarding various aspects of the program. The satisfaction scale demonstrated good internal consistency reliability, with a Cronbach's alpha value of 0.87. Face-to-face interviews and focus group discussions (FGDs) were conducted using open-ended questionnaires to gather qualitative data and insights from participants regarding their experiences with the VHVs' abilities program. The four open-ended questions included: 1) “Do you feel confident when are you coaching medical students at home visits?” 2) “Do you think the training program would be effective in improving VHV skills for associate teachers?” 3) “Were you able to complete the activities in the training program you want to?” 4) “What did you like most and like least about the training program? Why?”

### Statistical analysis

Descriptive statistics were used for all variables. Categorical variables were presented as frequency and percentage, while continuous variables were described using mean ± standard deviation and median with range. The normality of each subscale was assessed using the Shapiro-Wilk and Shapiro-Francia tests. The mean change over time of VHVs' competency scale was analyzed using a one-group repeated-measures analysis of variance (ANOVA).  Statistical significance was determined using two-sided analyses with a significance level set at p-value < 0.05. All statistical analyses were performed using STATA version 14.0.

The qualitative data obtained from face-to-face interviews and FGDs were analyzed using a conventional approach to content analysis.18 The data obtained from interviews and FGDs were transcribed, and the transcripts were carefully reviewed. Meaningful units, such as phrases, sentences, or paragraphs, were identified and assigned descriptive codes. The labeled units were then grouped into subcategories and categories through a process of comparative analysis. The categories and subcategories, along with the extracted codes, were examined and reviewed by the researchers (KK and SR). The final categories and subcategories were established, representing the key themes and findings derived from the qualitative data analysis.

## Results

Out of the 100 VHVs who participated in the VHVs’ abilities program, the majority were female, with an average age of 59.31 ± 10.06 years. 83 VHVs, equivalent to 83%, responded to the attitude, motivational, and satisfaction questionnaires before and after completing the program. Most participants were in agriculture, completed junior high school, and married. Participants have a median salary of 5,000 THB per month. Most participants were healthy, with an average body mass index of 25.58 ± 4.56 Kg/m2. Most participants were VHVs with over 10 years of experience. Only 40% of participants have a computer/smartphone literate (Table 1).

Before and after participation in the VHVs' abilities program, scores for the VHVs' competency scale to promote engaged community-based interprofessional education (CBIPE) field practice for medical students were assessed. A repeated-measures ANOVA revealed a significant difference in the five domains of the VHVs' competency scale after the program compared to before (Table 2).

**Table 1:** Demographic Characteristics of VHVs (N=83)

Demographics		N	%
Gender			
	Female	67	80.72
	Male	16	19.28
Educational background			
	Junior high school	46	55.42
	Senior high school/Vocational certificate	29	34.94
	Diploma/High vocational certificate	5	6.02
	Bachelor’s degree	3	3.61
Main occupation			
	Agriculture	30	36.14
	Housekeeper	26	31.33
	Employee	18	21.69
	Unemployed	4	4.82
	Government employee	4	4.82
	Company employee	1	1.20
Marital status			
	Marriage	59	71.08
	Divorce/Separate/Widow	14	16.87
	Single	10	12.05
Monthly income (THB per month), Median (IQR)		5,000	(600-50,000)
Health Status			
	Healthy	36	43.37
	Hypertension (HT)	14	16.86
	HT, DM, DLP	12	14.46
	HT, DM	6	7.23
	Diabetes Mellitus (DM)	4	4.82
	Dyslipidemia (DLP)	4	4.82
	HT, DLP	3	3.61
	DM, DLP	1	1.20
	DM, Thyroid	1	1.20
	Anemia	1	1.20
	Asthma	1	1.20
Body mass index (kg/m^2^), Mean (SD)		25.58	(4.65)
Years of experience in VHV			
	<5	9	10.84
	5-10	19	22.89
	10+	55	66.27
Skills and Abilities			
	Active listening skills	75	90.36
	Proficient oral communication	70	84.34
	Strong reading comprehension	70	84.34
	Effective written communication	69	83.13
	Computer/smartphone literate	33	39.76

After 1 week of training, there was a significant increase of 1 point in the attitude towards associate teachers (mean difference=1.00, 95%CI=0.96-1.04, P<0.001), motivation in the role of VHV (mean difference=0.92, 95%CI=0.86-0.97, P<0.001), communication (mean difference=0.95, 95%CI=0.92-0.97, P<0.001), and system thinking scale (mean difference=0.98, 95%CI=0.97-1.00, P<0.001). After 12 weeks of training, there was a significant slight increase only in motivation in the role of VHV (mean difference=0.08, 95%CI=0.03-0.14, P=0.004), communication (mean difference=0.05, 95%CI=0.03-0.08, P<0.001), and system thinking scale (mean difference=0.02, 95%CI=0.01-0.03, P<0.001).

In addition, a significant increase in the community diagnosis knowledge scale was observed after initiating the program (mean difference=26, 95%CI=24-28, P<0.001).

**Table 2:** Repeated measures ANOVA presented as mean change over time of village health volunteers' competency scale

Competency scale	Time(week)	Mean ± SD	p-value	Multiple comparisons	
				Time	Mean diff. (95%CI), p-value
Attitude	0	3.45 ± 0.50	<0.001	1 vs 0	1 (0.96-1.04), <0.001
	1	4.45 ± 0.50		12 vs 0	1 (0.96-1.04), <0.001
	12	4.45 ± 0.50		12 vs 1	0 (-0.04-0.04), 1.000
Motivation	0	3.35 ± 0.63	<0.001	1 vs 0	0.92 (0.86-0.97), <0.001
	1	4.24 ± 0.60		12 vs 0	1 (0.94-1.06), <0.001
	12	4.32 ± 0.62		12 vs 1	0.08 (0.03-0.14), 0.004
Active listening	0	3.85 ± 0.67	0.0628	1 vs 0	0.07 (-0.01-0.14), 0.104
	1	3.92 ± 0.65		12 vs 0	0.09 (-0.01-0.17), 0.052
	12	3.95 ± 0.64		12 vs 1	0.03 (-0.05-0.11), 0.504
Communication	0	3.05 ± 0.60	<0.001	1 vs 0	0.95 (0.92-0.97), <0.001
	1	4.00 ± 0.63		12 vs 0	1 (0.97-1.02), <0.001
	12	4.05 ± 0.60		12 vs 1	0.05 (0.03-0.08), <0.001
System thinking	0	2.98 ± 0.62	<0.001	1 vs 0	0.98 (0.97-1.00), <0.001
	1	3.97 ± 0.63		12 vs 0	1 (0.98-1.00), <0.001
	12	3.98 ± 0.62		12 vs 1	0.02 (0.01-0.03), <0.001
Knowledge	0	59.45 ± 6.14	<0.001	1 vs 0	25.71 (23.56-27.86), <0.001
	1^*^	85.15 ± 7.96		12 vs 0	17.66 (15.51-19.81), <0.001
	12	77.10 ± 8.00		12 vs 1	-8.05 (-5.89 - -10.20), <0.001

*Mean value at day 2 (after initiating the program)

**Table 3:** Distribution of VHVs satisfaction in the training program

Items	Mean	SD	95%CI of mean	Satisfied levels
1. Skills development	3.52	1.03	3.22-3.82	Satisfied
2. Infrastructure and support	3.72	1.04	3.42-4.02	Satisfied
3. Perceived quality of the advisor	3.77	0.95	3.50-4.05	Satisfied
4. Overall satisfaction	4.13	0.76	3.90-4.35	Satisfied

SD = standard deviation

However, after 12 weeks of training, the community diagnosis knowledge scale decreased significantly (mean difference=-8, 95%CI=-6 - -10, P<0.001). On the contrary, There were no significant changes in the active listening scale throughout the research period. The VHVs' satisfaction with the VHVs' abilities program was assessed, revealing that most VHVs were satisfied with the program. The average satisfaction score was 4.13 ± 0.76 out of 5 points (Table 3).

Most VHVs openly reflected and explained that the VHVs’ abilities program helped them fill their knowledge gaps and improved the VHVs competency skills including improved competency awareness in communication, and system thinking, understanding of the roles of associate teachers, and enhanced understanding of community diagnosis within the collaborative practice. A participant from VHVs commented:

"It's been such a rewarding experience for me. Even though I only completed secondary school, the training program and guidance from the professors have given me so much confidence. Now, I feel like I can truly make a difference and help not only the medical students but also others in our community. I'm grateful for the opportunity to learn and grow through this program." (Female VHV, aged 58 years)

"The training program has been incredibly valuable for me. It has enhanced my understanding of community diagnosis and has greatly improved my competency awareness as an associate teacher. Moreover, it has sharpened my communication skills and provided clarity on my roles within our collaborative practice. I'm grateful for the knowledge and skills I've gained through this program." (Male VHV, aged 62 years)

"This program has truly made me feel useful and empowered. I've noticed significant improvements in my communication skills, which have been invaluable during our interactions with medical students. However, I still hope to further develop my comfort level when discussing community contexts with the students. Overall, I'm grateful for the opportunity to participate in this program and for the positive impact it has had on my abilities and confidence." (Male VHV, aged 54 years)

"I am thrilled to have participated in this workshop. It has been an enriching experience for me, and I now feel much more confident and prepared to coach medical students during home visits. The knowledge and skills I have gained through this workshop have equipped me to make a meaningful difference in the education and training of future healthcare professionals. I am grateful for the opportunity and look forward to applying what I have learned in my interactions with medical students." (Female VHV, aged 68 years)

"I strongly believe that this program should continue because of the immense benefits it provides to VHVs in improving their knowledge and skills. I can attest to the positive impact it has had on me. Since completing the program, I feel much more confident and prepared to coach medical students during home visits. This program has been instrumental in enhancing our abilities as VHVs, and I hope it continues to be offered to empower other volunteers like myself." (Female VHV, aged 65 years)

"I have always been dedicated to taking care of medical students and providing them with relevant health information and community context in my community. However, there have been times when I felt unable to fully carry out my duties because I struggled to explain the links between diseases and provide proper guidance. Thankfully, after participating in a 2-day intensive training program with my fellow VHVs, I gained valuable insights into how to effectively communicate and discuss disease links and community context with the medical students. This program has equipped me with the necessary skills and confidence to fulfill my responsibilities more effectively." (Male VHV, aged 54 years)

However, one of the limitations of the VHVs' abilities program was the inadequate duration allocated for brief active listening skills training.

"It brings me immense joy and confidence to be part of the associate teachers' team. I am convinced that every VHV, like myself, feels more confident and prepared to coach medical students during home visits. However, it's unfortunate that there isn't enough time dedicated to improving active listening skills. This aspect is crucial for effective communication and understanding, and I believe allocating more time to enhance these skills would greatly benefit both VHVs and medical students." (Female VHV, aged 50 years)

"The 2-day intensive program offered exceptional opportunities to develop VHV professional skills in a real-world setting. However, I felt that the program lacked sufficient time to focus on improving my active listening skills. This skill is vital for effective communication and understanding, and I believe more dedicated time to hone this ability would have been beneficial." (Male VHV, aged 64 years)

## Discussion

Previous studies have shown that the CBIPE program is designed to enhance the collaborative abilities of medical students and community members. CBIPE programs provide opportunities for active engagement of both medical students and community members in various learning activities throughout the educational experience and practical fieldwork. 5-10 The previous studies demonstrate that the CBIPE program helps students gain experience by working within the community and conducting community diagnosis through polite discussions with patients and VHVs.^12^^,^^19^ Strengthening VHVs' knowledge, soft skills, and self-confidence can significantly enhance their ability to support CBIPE initiatives. VHVs with enhanced abilities can also play a pivotal role in promoting interprofessional collaborative competencies among medical students. Therefore, implementing a program to increase the abilities of VHVs can have several potential benefits in promoting engaged CBIPE field practice for medical students. Overall, increasing the abilities of VHVs through targeted programs can be an effective strategy for promoting engaged CBIPE field practice for medical students. It offers valuable opportunities for experiential learning, interprofessional collaboration, cultural competence development, and community engagement, ultimately preparing students to become competent and compassionate healthcare professionals.

In this study, the VHVs were likely to be older adults, established, and respected in the community, despite having primary education. The present results have shown that the five domains of VHVs competence skills had a significant difference between the measurements before and after the VHVs’ abilities program. The program was found to be feasible and effective in enhancing the knowledge, attitude, and motivation of VHVs, as well as improving their communication and systems thinking skills. This indicates that investing in training programs for VHVs can lead to significant improvements in their abilities. The finding also found that while there was an initial increase in community diagnosis knowledge after the program, this knowledge started to decrease after 12 weeks. This highlights the need for ongoing and regular training sessions, particularly focused on community diagnosis, to maintain and reinforce knowledge over time. Identifying and developing core program activities based on community diagnosis was a challenge. The design and development of the program should be guided by principles of community-based participatory research (CBPR). Key stakeholders, including VHVs themselves, should be actively involved in the design and development of the program. Their input is crucial for ensuring that the program is tailored to address the specific needs, resources, and cultural context of the community. Creating a supportive environment is essential for the success of the program. This can include providing resources such as handbooks, as well as offering supervision and feedback to VHVs. Regular feedback sessions with stakeholders can help ensure that the program activities align with their expectations and overcome any barriers that may arise. The program should be dynamic and responsive to feedback and changing community needs. Regular evaluations and assessments can help identify areas for improvement and ensure that the program remains effective and relevant over time. Moreover, online training sessions with various interactive components, including a mini-lecture, video demonstration, remote practice in pairs remotely overseen by the mentors, and feedback, may be holds great potential for enhancing the skills and knowledge of VHVs. Due to online teaching based on computer networks offers numerous advantages that enhance accessibility, flexibility, convenience, and effectiveness in education.^20^^-^^23^ In addition, network teaching platforms offer numerous advantages over traditional onsite teaching models, including increased accessibility, scalability, flexibility, customization, interactivity, and efficiency.^24^ Previous studies have shown that online learning has the potential to significantly enhance the quality of higher education by offering opportunities for lifelong learning and professional development.^23^^,^^25^^,^^26^ The present study also revealed that most of the VHVs were satisfied with the VHVs’ abilities program. Most VHVs demonstrated that the program improved their awareness of their abilities in communication, system thinking, understanding the roles of associate teachers, and community diagnosis. This increased awareness likely contributed to their confidence and effectiveness in supporting CBIPE activities.

The key factors of the program included a supportive environment, participatory learning, demonstration and replay demonstration, case discussion and practice, information sharing and communication, and activities with feedback. Creating a supportive environment was crucial for the success of the program. This included providing encouragement, resources, and feedback to VHVs, fostering a sense of belonging and empowerment. The program adopted a participatory learning approach, actively involving VHVs in the learning process. This hands-on approach allowed VHVs to apply their knowledge and skills in real-world scenarios, reinforcing their learning and building confidence. Utilizing demonstrations and replay demonstrations helped reinforce key concepts and techniques. VHVs could observe and practice skills, enhancing their understanding and proficiency. Engaging VHVs in case discussions and practical exercises allowed them to apply their knowledge to real-life situations. This practical experience was instrumental in building their confidence and competence. Open communication and information sharing were encouraged throughout the program. This facilitated collaboration among VHVs and other stakeholders, fostering a sense of community and shared purpose. Providing regular feedback allowed VHVs to track their progress and identify areas for improvement. Small-group discussions provided a forum for VHVs to share ideas and experiences, enhancing their awareness and understanding. These discussions fostered a sense of camaraderie and mutual support among participants. Therefore, the heightened small group discussion and communication within a learning activity can effectively increase awareness.

To facilitate collaborative and effective communication, the importance of active listening skills and being open-minded were recognized by VHVs. They described how listening skills might enhance communication and improve care when they functioned as a team. They emphasized that each person could make valuable contributions and that being open to other people’s opinions was important. On the contrary, the active listening scale had a small change. It is assumed that the program had only a small effect on enhancing abilities. Most VHVs reflected that the program had limitations, particularly in terms of insufficient time for improvement of their active learning skills. Improving active listening skills remains a challenge. An online training program demonstrated that brief active listening skills training was associated with significant improvements in active listening skills.^27^^,^^28^ Active listening is a fundamental skill for effective communication and can significantly impact patient-provider interactions and outcomes. Prioritizing the enhancement of these skills for VHVs is crucial, as they play a key role in delivering healthcare services and promoting health within their communities. However, it's essential to recognize that the effectiveness and acceptability of active listening training among VHVs may vary depending on factors such as cultural context, existing knowledge and skills, and individual learning preferences. Therefore, further study is warranted to explore the longer-term effectiveness and acceptability of such training programs specifically tailored for VHVs.

### Limitations

Our present study had some limitations. A single-group, non-randomization with a pre-post-posttest design did not have a comparison group, which is an essential component of any study. Future studies could incorporate randomized controlled trials (RCTs) with control groups to assess the program's impact more rigorously. On the other hand, the Hawthorne effect, where participants modify their behavior due to awareness of being observed, could have influenced the results. To minimize this effect, researchers could consider blinding participants to the intervention or using control groups to provide a baseline for comparison. Non-respondents may differ from respondents, potentially leading to overestimation of the program's effectiveness. Strategies such as increasing outreach efforts, incentivizing participation, and ensuring confidentiality may help mitigate non-response bias. The low baseline scores of knowledge and skills could have inflated the perceived effectiveness of the program. Future studies could include participants with a wider range of baseline abilities to provide a more accurate assessment of the program's impact.

## Conclusions

In conclusion, the present study showed that well-designed training programs for VHVs can lead to improved knowledge, communication skills, systems thinking, confidence to support CBIPE, and better engagement with medical students. These programs also help VHVs become more effective in their roles as associate teachers and community health promoters. Strengthening the program through future research, such as RCTs, would provide a rigorous evaluation of its effectiveness. Extending the program's duration and implementing longer follow-up periods would assess its sustainability and long-term benefits on VHVs' community performance. These steps would yield valuable insights into the program's impact and its enduring benefits for VHVs in the community.

The positive outcomes of VHVs' training programs underscore the significance of integrating similar modules into medical education curricula. This integration ensures that future healthcare professionals are proficient in communication, systems thinking, and community diagnosis. Including community engagement in medical education offers students practical experience and enhances their comprehension of community health needs. Emphasizing soft skills development—such as communication and teamwork—is essential for effective healthcare delivery and should be integral to medical education. Research into interprofessional education initiatives involving VHVs, medical students, and other healthcare professionals can elucidate the benefits of teamwork and integrated care approaches.

### Acknowledgments

The authors extend their gratitude to all the teaching staff and village health volunteers who generously contributed to this research study. Additionally, special thanks are owed to the Department of Preventive and Social Medicine, Faculty of Medicine, Srinakharinwirot University, for providing the invaluable opportunity to conduct this study.

### Conflict of Interest

The author declares that there is no conflict of interest.
